# Historical Prediction Modeling Approach for Estimating Long-Term Concentrations of PM_2.5_ in Cohort Studies before the 1999 Implementation of Widespread Monitoring

**DOI:** 10.1289/EHP131

**Published:** 2016-06-24

**Authors:** Sun-Young Kim, Casey Olives, Lianne Sheppard, Paul D. Sampson, Timothy V. Larson, Joshua P. Keller, Joel D. Kaufman

**Affiliations:** 1 Institute of Health and Environment, Seoul National University, Seoul, Korea; 2 Department of Environmental and Occupational Health Sciences,; 3 Department of Biostatistics,; 4 Department of Statistics,; 5 Department of Civil and Environmental Engineering,; 6 Department of Epidemiology, and; 7 Department of Medicine, University of Washington, Seattle, Seattle, Washington, USA

## Abstract

**Introduction:**

Recent cohort studies have used exposure prediction models to estimate the association between long-term residential concentrations of fine particulate matter (PM2.5) and health. Because these prediction models rely on PM2.5 monitoring data, predictions for times before extensive spatial monitoring present a challenge to understanding long-term exposure effects. The U.S. Environmental Protection Agency (EPA) Federal Reference Method (FRM) network for PM2.5 was established in 1999.

**Objectives:**

We evaluated a novel statistical approach to produce high-quality exposure predictions from 1980 through 2010 in the continental United States for epidemiological applications.

**Methods:**

We developed spatio-temporal prediction models using geographic predictors and annual average PM2.5 data from 1999 through 2010 from the FRM and the Interagency Monitoring of Protected Visual Environments (IMPROVE) networks. Temporal trends before 1999 were estimated by using a) extrapolation based on PM2.5 data in FRM/IMPROVE, b) PM2.5 sulfate data in the Clean Air Status and Trends Network, and c) visibility data across the Weather Bureau Army Navy network. We validated the models using PM2.5 data collected before 1999 from IMPROVE, California Air Resources Board dichotomous sampler monitoring (CARB dichot), the Children’s Health Study (CHS), and the Inhalable Particulate Network (IPN).

**Results:**

In our validation using pre-1999 data, the prediction model performed well across three trend estimation approaches when validated using IMPROVE and CHS data (R2 = 0.84–0.91) with lower R2 values in early years. Model performance using CARB dichot and IPN data was worse (R2 = 0.00–0.85) most likely because of fewer monitoring sites and inconsistent sampling methods.

**Conclusions:**

Our prediction modeling approach will allow health effects estimation associated with long-term exposures to PM2.5 over extended time periods ≤ 30 years.

**Citation:**

Kim SY, Olives C, Sheppard L, Sampson PD, Larson TV, Keller JP, Kaufman JD. 2017. Historical prediction modeling approach for estimating long-term concentrations of PM2.5 in cohort studies before the 1999 implementation of widespread monitoring. Environ Health Perspect 125:38–46; http://dx.doi.org/10.1289/EHP131

## Introduction

Many cohort studies of the long-term effects of fine particulate matter (PM_2.5_) air pollution on health have used exposure prediction models to estimate individual-level long-term concentrations at cohort residences (e.g., Beelen et al. 2014; Eeftens et al. 2012; Paciorek et al. 2009; Puett et al. 2009; Sampson et al. 2013; Young et al. 2014). These exposure prediction models rely on PM_2.5_ monitoring data collected from spatially distributed monitoring networks. PM_2.5_ predictions are generally infeasible for times before comprehensive spatial monitoring began (in the late 1990s or 2000s, depending on the country). However, many cohorts were enrolled before these extensive monitoring networks began operating. Therefore, many studies use PM_2.5_ estimates based on monitoring data from later time periods than cohort follow-up for their health analyses (e.g., Beelen et al. 2008; Cesaroni et al. 2013; Weichenthal et al. 2014). This temporal misalignment of PM_2.5_ predictions with health data could affect study results.

Other studies have developed historical prediction models to temporally align exposure estimates with health outcomes. These studies used back-extrapolation, historically available large-size particle data, or physical or chemical models complemented by visibility, emission, meteorology, and satellite data (Beelen et al. 2014; Brauer et al. 2012; Hogrefe et al. 2009; Hystad et al. 2012; Lall et al. 2004; Molnár et al. 2015; Ozkaynak et al. 1985; Paciorek et al. 2009; Yanosky et al. 2009). However, most of these studies estimated historical PM_2.5_ concentrations in limited areas or for relatively short time periods, or for a combination of the two. Furthermore, the model evaluation for the period before extensive monitoring was restricted to small data sets or was poorly reported.

In the United States, many populations of great value for assessment of PM_2.5_ health effects collected data well before 1999, when reliable long-term regulatory monitoring data for PM_2.5_ began to be available. We aimed to develop a national prediction model to estimate annual average concentrations of PM_2.5_ in the continental United States for the entire time period from 1980 through 2010. We evaluated our historical predictions from 1980 through 1998 using available external validation data sets and investigated residential historical predictions using a multicity cohort.

## Methods

### PM_2.5_ Data

**Figure 1:**
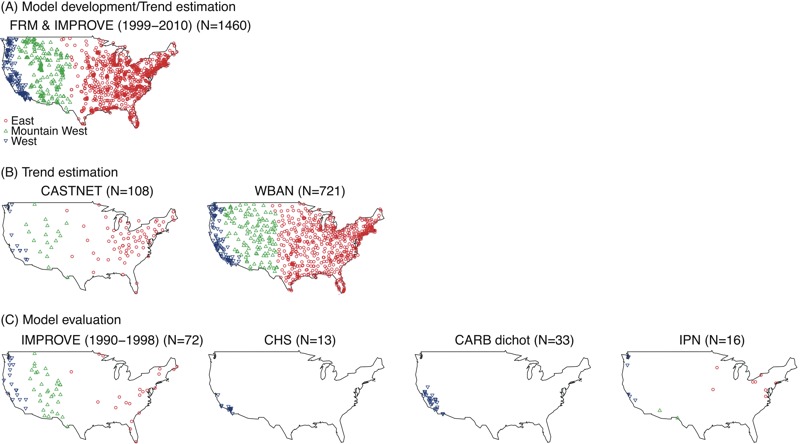
Maps of (*A*) FRM and IMPROVE sites for 1999–2010 used in model development and trend estimation, (*B*) CASTNet and WBAN sites used for trend estimation, and (*C*) IMPROVE sites for 1990–1998, CHS, CARB dichot, and IPN sites used in model evaluation (blue, green, and red symbols represent West, Mountain West, and East regions, respectively); Maps generated using locations of regulatory monitoring sites downloaded from the U.S. Environmental Protection Agency (EPA) website (http://aqsdr1.epa.gov/aqsweb/aqstmp/airdata/download_files.html#Daily) and boundaries in the R package (version 3.2.5; R Project for Statistical Computing). CARB dichot, California Air Resources Board dichotomous sampler monitoring; CASTNet, Clean Air Status and Trends Network; CHS, Children’s Health Study; FRM, Federal Reference Method; IMPROVE, Interagency Monitoring of Protected Visual Environment; IPN, Inhalable Particulate Network; WBAN, Weather Bureau Army Navy.

We obtained daily PM_2.5_ concentrations from the two national PM_2.5_ monitoring networks: the U.S. Environmental Protection Agency (EPA) Federal Reference Method (FRM) network and the Interagency Monitoring of Protected Visual Environment (IMPROVE) network. Whereas FRM sites were located mostly in urban areas to monitor population-level PM_2.5_ concentrations, IMPROVE sites were established to monitor visibility and were located mostly in wilderness areas and national parks (Hand et al. 2011; U.S. EPA 2004b). We downloaded all available data from FRM (1999 through 2010) and IMPROVE sites (1990 through 2010) from the U.S. EPA Air Quality database (U.S. EPA 2014). We computed annual averages of PM_2.5_ for each site that met the minimum inclusion criteria of at least two-thirds complete data points for any year (with exact numbers dependent on the sampling schedule) and < 45 consecutive missing days of sampling. We used the PM_2.5_ data collected from the FRM and IMPROVE networks for 1999–2010 for model development including temporal trend estimation, whereas we reserved the IMPROVE data from 1990 through 1998 for model validation. We categorized all monitoring sites into three regions: East, Mountain West, and West Coast (Figure 1).

To estimate temporal trends for the entire time period from 1980 through 2010, including all years without FRM PM_2.5_ measurements, we obtained two additional sources of data: annual average concentrations of PM_2.5_ sulfate measured in the Clean Air Status and Trends Network (CASTNet) from 1987 through 2010 (U.S. EPA 2015) and daily noon-time visual ranges, as a measure of visibility, monitored in the Weather Bureau Army Navy (WBAN) network from 1980 through 2010. Because most visibility measurements collected by optical instruments had a maximum of 16.093 km (10 mi), and because the use of these instruments replaced taking measurements with the human eye in the 1990s (U.S. EPA 2005), we truncated all measurements to a maximum distance of 16.093 km. We computed annual averages of visibility after excluding days with heavy fog, dust, and precipitation, and after applying the same inclusion criteria as those used for PM_2.5_ data.

For model evaluation in years prior to 1999, we obtained PM_2.5_ data from three different networks in addition to IMPROVE: the Southern California Children’s Health Study (CHS) for 1988–2001 (Peters et al. 2004), the California Air Resources Board dichotomous sampler monitoring (CARB dichot) for 1994–2003 in California (Blanchard et al. 2011), and the Inhalable Particulate Network (IPN) for 1979–1982 over the continental United States (Hinton et al. 1985). CHS PM_2.5_ data collected using 2-week samplers were converted to FRM-equivalent PM_2.5_ data for computing annual averages (Peters et al. 2004). Likewise, for the CARB dichot data, we adopted a published conversion equation to estimate FRM-equivalent PM_2.5_ (Blanchard et al. 2011). We applied the same inclusion criteria to sites in the three model evaluation networks to compute annual averages. These criteria reduced the number of IPN sites from 102 (for 1979–1982) to 16 (for 1980–1981), whereas the other three networks yielded the same or consistent numbers of sites.

### Geographic Variables and Geocoding

We considered > 800 variables representing geographic characteristics including traffic, land use, emission, elevation, and vegetation index (see Table S1). Computation of these variables at each of the PM_2.5_ monitoring sites was implemented in ArcGIS 10.2. For land use characteristics, we used data collected during different time periods to incorporate time-varying spatial features into the model: land cover data from the 1970s and 1980s and satellite land use imagery data generated in 2006. Our final list of geographic variables was pruned to ~300 variables after we eliminated the less-informative variables with little variability. To illustrate our predictions over time, we geocoded the residential addresses of 7,552 participants in the Multi-Ethnic Study of Atherosclerosis (MESA) (Bild et al. 2002) and the associated MESA Air project (Kaufman et al. 2012). These participants provided historical residential addresses dating back to 1980. In addition, we generated the coordinates of 12,501 points on a 25-km grid across the continental United States.

### Development of the PM_2.5_ Model for 1980–2010

The PM_2.5_ model for the period of 1980–2010 was developed based on the framework of the PM_2.5_ spatio-temporal prediction model in MESA Air (Keller et al. 2015; Lindström et al. 2014; Sampson et al. 2011; Szpiro et al. 2009). Briefly, the MESA Air spatio-temporal prediction model analyzed 2-week averages of PM_2.5_ as a function of a spatially varying long-term mean, spatially varying temporal trends, and spatio-temporal residuals. The spatially varying temporal trends were composed of spatially varying trend coefficients and trend basis functions. The trend basis functions were estimated from singular value decomposition of the data from sites with long time series (Fuentes et al. 2006). The spatially varying long-term mean and trend coefficients were estimated using universal kriging, which integrates geographic predictors and spatial smoothing (Banerjee et al. 2004). Before regression modeling, we used partial least squares (PLS) to reduce the dimension of the hundreds of geographic variables to a limited number of derived predictors that were the linear combinations that maximized their covariance with PM_2.5_. The spatial dependence structure in the kriging model for the long-term mean was assumed to be exponential and was parameterized by three components: the range, partial sill, and nugget. The spatially dependent and temporally independent spatio-temporal residuals were modeled by using simple kriging. Whereas the MESA Air model was based on 2-week averages, in this work, we modeled the log(annual average PM_2.5_ concentrations) from 1999 through 2010. For the trend estimation, we considered only sites with > 6 years of monitoring out of the 12 possible years. To avoid unnecessary complexity in the model, we assumed a single temporal trend, no spatial structure for the trend coefficient (zero range and partial sill), and two PLS predictors. We examined alternative modeling choices by including a spatial structure for the trend coefficient and interaction terms for three regions.

We explored various approaches to estimating the temporal trend before 1999. These approaches included backward extrapolation of the temporal trend basis function estimated from the 1999–2010 FRM PM_2.5_ data and estimation of the temporal trend using other sources of data such as emissions, meteorological variables, visibility, and PM_2.5_ sulfate; all of these other measurements have been shown to be associated with PM_2.5_ in previous studies (Hand et al. 2014; Malm et al. 2002; Ozkaynak et al. 1985). Ultimately, we selected three approaches for in-depth evaluation of the historical trend estimation: *a*) extrapolation of the linear trend estimated on the basis of the PM_2.5_ data in FRM and IMPROVE for 1999–2010; *b*) estimation of the trend using the PM_2.5_ sulfate data in CASTNet for 1987–2010 and extrapolation for 1980–1986; and *c*) estimation of the trend using the visibility data in WBAN for 1980–2010. We also examined alternative approaches, including combining two data sources into one temporal trend, estimating two temporal trends, and replacing the trend with meteorological variables as spatio-temporal covariates.

To evaluate our model for 1999–2010, we performed 5-fold cross-validation and computed the root mean square error (RMSE) and MSE-based *R*-square (*R* ^2^) statistics for the annual averages (Keller et al. 2015). The MSE-based *R* ^2^ was calculated by subtracting from 1 the ratio of the MSE to the variance of the data. This value evaluates predictions compared with observations about the identity line. In contrast, traditional regression-based *R* ^2^, the squared correlation coefficient, compares predictions with observations about a regression line, which can result in overestimation of prediction ability. We presented cross-validation statistics for each year and for all 12 years combined for all sites, and for all 12 years combined within each of the three regions. In addition to spatial performance, we examined temporal performance by using the median of the cross-validation statistics at each site for which > 6 years of data were available. To aid in assessing bias, we have also provided slopes and intercepts from the regression of cross-validated predictions on observations (see Supplemental Material).

### Model Evaluation for the Pre-1999 Period

We externally validated the model using four distinct PM_2.5_ data sets, all of which were sampled before 1999: *a*) IMPROVE data for 1990–1998, *b*) CARB dichot data for 1988–2001, *c*) CHS data for 1994–2003, and *d*) IPN data for 1980–1981 (Table 1). We predicted annual averages of PM_2.5_ concentrations at monitoring sites in each of the four monitoring networks and computed out-of-sample RMSEs and MSE-based *R* ^2^s using these external data sources for all years and regions as well as by year and region. We also estimated the intercepts and slopes of the best-fit lines.

**Table 1:** Summary of PM_2.5_ monitoring data used for PM_2.5_ historical model development and validation.

Network	Spatial coverage	Regulatory monitoring network	Number of sites^*a*^	Number of observations^*a*^	Sampling period^*a*^	Annual average of PM_2.5_ (μg/m^3^) Mean ± SD
FRM	National (urban)	Yes	1,282	9,233	1999–2010	12.03 ± 3.23
IMPROVE	National (rural)	Yes	178	1,567	1999–2010	5.44 ± 2.94
			72	423	1990–1998	6.05 ± 3.75
CASTNet	National (rural)	Yes	108	1,485	1987–2010	3.15 ± 1.91
IPN	National (urban/rural)	Yes	16	18	1980–1981	21.31 ± 6.69
CARB dichot	California (urban/rural)	Yes	33	247	1988–2001	19.35 ± 7.78
CHS	Southern California (urban)	No	13	120	1994–2003	16.12 ± 8.17
Notes: CARB dichot, California Air Resources Board dichotomous sampler monitoring; CASTNet, Clean Air Status and Trends Network; CHS, Children’s Health Study; FRM, Federal Reference Method; IMPROVE, Interagency Monitoring of Protected Visual Environment; IPN, Inhalable Particulate Network; PM_2.5_, fine particulate matter. ^***a***^Number of sites, number of observations, and sampling period for the monitoring sites that meet the minimum inclusion criteria for computing representative annual averages.						

### Predictions

We created maps of PM_2.5_ predictions on a 25-km grid over the contiguous United States for 1980, 1990, 2000, and 2010 to examine spatially varying changes of PM_2.5_ concentrations over time. We also selected the 10 grid coordinates with the highest populations in each of the three regions and explored the trends of the predictions over 31 years.

In addition, we conducted analyses to provide information on the degree to which exposure estimation based on data from the year 2000 reflected concentrations predicted by our approach in the earlier period. To investigate the sensitivity of individual exposure estimates to temporal and spatial variation resulting from changes in people’s residences over time, we predicted PM_2.5_ concentrations at all home addresses from 1980 through 2000, the year of the baseline exam, among members of the MESA/MESA Air cohort and computed a 21-year average weighted by residence times across historical addresses for each participant. These predictions were compared with annual averages estimated for the same participants in 2000, the year of the baseline exam. We stratified this comparison by the 5,086 participants who did not move during 1980–2000 (“nonmovers”) and the 2,466 people who moved at least once.

## Results

**Figure 2:**
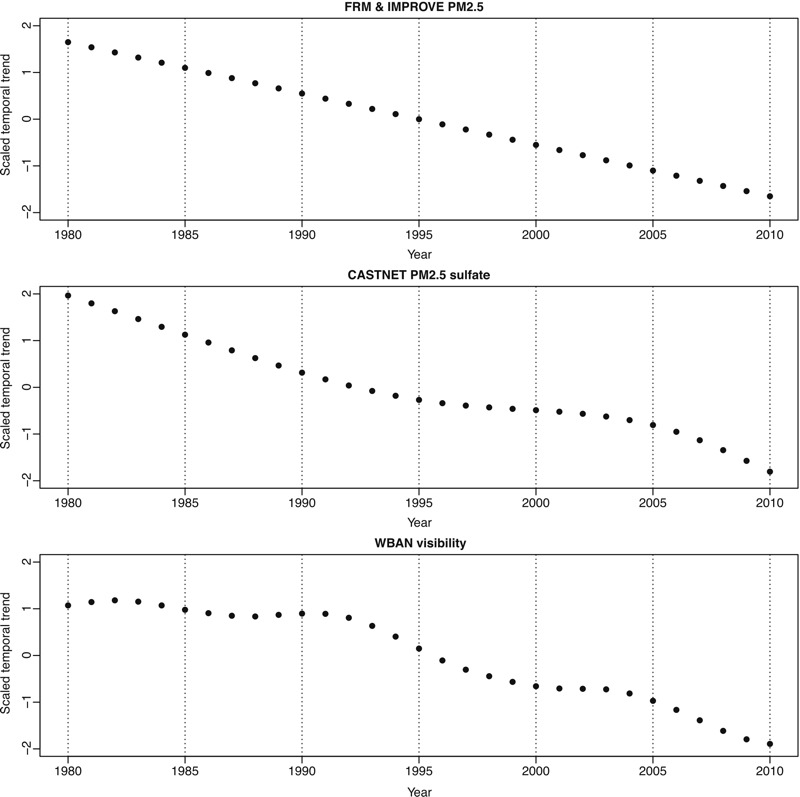
Estimated temporal trends based on fine particulate matter (PM_2.5_) annual averages in FRM and IMPROVE, PM_2.5_ sulfate annual averages in CASTNet, and visibility annual averages in WBAN. Notes: CASTNet, Clean Air Status and Trends Network; FRM, Federal Reference Method; IMPROVE, Interagency Monitoring of Protected Visual Environment; WBAN, Weather Bureau Army Navy.

The means of PM_2.5_ annual averages for 1999–2010 from FRM and IMPROVE were 12.03 (SD = 3.23) and 5.44 (2.94) μg/m^3^, respectively (Table 1). There were far fewer monitoring sites in 1999 than in 2000–2010 (see Figure S1), and most of the 1999–2010 sites were located in the East region (Figure 1). The annual average concentrations of PM_2.5_ decreased over time from 1999 through 2010, particularly in the East and West Coast regions (see Figure S2). Figure 2 shows the estimated temporal trends from 1980 through 2010 using the three trend estimation approaches described in “Methods.” Whereas the extrapolated trend based on the PM_2.5_ data was linear, the trends estimated using PM_2.5_ sulfate and visibility measurements had different rates of decrease in different time periods with approximate linearity over time.

In the model evaluation for 1999–2010, cross-validated *R* ^2^s for all 12 years combined and each single year were high, varying between 0.77 and 0.87 across the three trend estimation approaches (see Tables S2 and S3). Temporally characterized *R* ^2^s at each site over years were lower (0.55–0.58) than spatially characterized *R* ^2^s in each year across sites, possibly because of relatively small temporal variability for 12 years compared with large spatial variability across the United States. The cross-validation statistics for the alternative modeling approaches in the sensitivity analyses were consistent with (and no better than) or poorer than those of our primary approach shown in Table S2 (data not shown).

Figure S3 shows estimated regression and variance parameters for the long-term mean, the temporal trend coefficient, and spatio-temporal residuals, and Figure S4 displays loadings of geographic variables for each PLS predictor. The regression coefficients of the two PLS predictors for both the long-term mean and the trend coefficient were statistically significantly different from 0, reflecting that spatial variation in the long-term mean and the temporal trend can be explained by the geographic variables used to create the PLS predictors. Significant range and partial sill parameters for the long-term mean indicate an additional important contribution of the spatial correlation structure to the long-term mean. The contribution of the temporal trend to the cross-validated predictions was smaller than that of the long-term mean (see Table S4).

**Figure 3:**
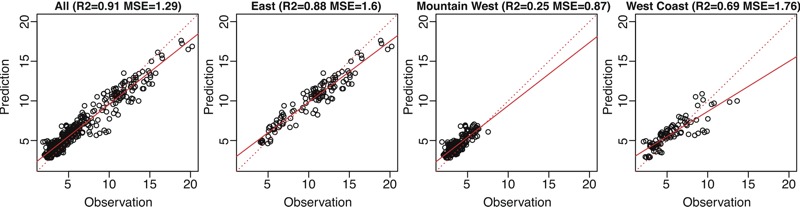
Scatter plots of observed and predicted fine particulate matter (PM_2.5_) annual averages from the PM_2.5_ historical model using the Federal Reference Method/Interagency Monitoring of Protected Visual Environment (FRM/IMPROVE) PM_2.5_ trend across IMPROVE sites for 1990–1998.

Tables 2 and 3 show the external validation statistics for the pre-1999 period using IMPROVE data and CHS, CARB dichot, and IPN data, respectively. Using IMPROVE data, the *R* ^2^ values were consistently high for all years and for each year separately (0.70–0.91) across the three trend estimation approaches (Table 2, Figure 3). The *R* ^2^ values were slightly higher for the model using the extrapolated linear trend based on PM_2.5_ data than the model using estimated trends from PM_2.5_ sulfate and visibility data. In addition, the earliest years (1990 and 1991) yielded lower *R* ^2^s (0.70–0.85) than the other years (0.83–0.93). The East region produced higher *R* ^2^s (0.67–0.88) than the Mountain West region. When the model was validated using the CHS data, the *R* ^2^ values were also generally high (0.71–0.90) (Table 3; see also Figure S5). The CARB dichot data yielded high *R* ^2^s (over 0.5) except for some years, whereas the IPN data consistently yielded low *R* ^2^s (Table 3; see also Figures S6 and S7). The variability of the predicted PM_2.5_ annual average concentrations tended to be smaller than that of the observations, with regressions on observations having slopes < 1 (see Tables S5 and S6). Figures S8 and S9 show the differences between the maximum and minimum predicted PM_2.5_ annual averages across three trend estimation approaches over years at IMPROVE sites. Median differences were small, and most were < 2 μg/m^3^. In addition, the differences were larger in the early years than in recent years, indicating increasing prediction uncertainty of trend estimation in the early years.

**Table 2:** External validation statistics of the historical PM_2.5_ models using PM_2.5_ IMPROVE data for 1990–1998 by estimated temporal trend, year, and region.

Year/region	*n*^*a*^	FRM/IMPROVE PM_2.5_		CASTNet PM_2.5_ sulfate		WBAN visibility	
		*R*^2^	RMSE (μg/m^3^)	*R*^2^	RMSE (μg/m^3^)	*R*^2^	RMSE (μg/m^3^)
All^*b*^	72 (423)	0.91	1.14	0.84	1.49	0.86	1.41
1990	30	0.85	1.04	0.78	1.26	0.70	1.48
1991	36	0.83	1.40	0.78	1.56	0.70	1.84
1992	37	0.91	1.19	0.84	1.59	0.85	1.57
1993	45	0.92	1.20	0.83	1.76	0.87	1.53
1994	50	0.92	1.03	0.84	1.45	0.89	1.20
1995	58	0.91	1.15	0.86	1.41	0.86	1.40
1996	56	0.93	0.93	0.88	1.26	0.91	1.10
1997	57	0.93	1.01	0.86	1.42	0.90	1.21
1998	54	0.90	1.28	0.83	1.70	0.87	1.46
East^*b*^	21 (120)	0.88	1.27	0.67	2.10	0.84	1.45
Mountain West^*b*^	34 (202)	0.25	0.93	0.04	1.06	0.00	1.39
West Coast^*b*^	17 (101)	0.69	1.33	0.67	1.37	0.66	1.39
Notes: CASTNet, Clean Air Status and Trends Network; FRM, Federal Reference Method; IMPROVE, Interagency Monitoring of Protected Visual Environment; PM_2.5_, fine particulate matter; RMSE, root mean square error; WBAN, Weather Bureau Army Navy. ^***a***^Number of sites (number of observations when different from the number of sites). ^***b***^Annual averages from 1990 through 1998.							

**Table 3:** External validation statistics of the historical PM_2.5_ models using CHS, CARB dichot, and IPN data by estimated temporal trend and year.

Validation data	Year	*n*^*a*^	FRM/IMPROVE**PM_2.5_		CASTNet PM_2.5_ sulfate		WBAN visibility	
			*R*^2^	RMSE (μg/m^3^)	*R*^2^	RMSE (μg/m^3^)	*R*^2^	RMSE (μg/m^3^)
CHS	All^*b*^	13 (120)	0.76	4.00	0.76	3.98	0.81	3.59
	1994	12	0.71	5.19	0.69	5.34	0.80	4.33
	1995	12	0.66	5.97	0.63	6.31	0.75	5.17
	1996	12	0.77	4.40	0.75	4.56	0.82	3.86
	1997	12	0.83	3.12	0.84	3.01	0.88	2.64
	1998	12	0.83	2.87	0.87	2.55	0.87	2.54
	1999	12	0.73	4.30	0.75	4.13	0.74	4.16
	2000	12	0.80	3.43	0.82	3.24	0.82	3.31
	2001	12	0.82	3.79	0.85	3.44	0.86	3.27
	2002	12	0.81	3.20	0.82	3.12	0.79	3.31
	2003	12	0.88	2.39	0.90	2.22	0.89	2.30
CARB dichot	All^*b*^	33 (162)	0.55	5.54	0.48	5.98	0.61	5.17
	1988	8	0.09	9.70	0.00	10.52	0.15	9.40
	1989	12	0.25	9.07	0.10	9.94	0.33	8.55
	1990	11	0.68	4.77	0.53	5.74	0.76	4.08
	1991	12	0.31	9.24	0.16	10.16	0.43	8.35
	1992	14	0.51	5.35	0.40	5.91	0.63	4.68
	1993	15	0.54	3.88	0.33	4.67	0.66	3.30
	1994	13	0.77	4.08	0.69	4.72	0.84	3.37
	1995	12	0.71	3.46	0.63	3.91	0.70	3.54
	1996	15	0.52	4.00	0.66	3.37	0.57	3.81
	1997	15	0.41	3.19	0.59	2.66	0.45	3.08
	1998	16	0.31	4.11	0.37	3.94	0.30	4.14
	1999	12	0.85	2.39	0.84	2.50	0.82	2.64
	2000	6	0.53	2.41	0.46	2.59	0.41	2.69
	2001	3	0.00	9.41	0.00	9.34	0.00	9.19
IPN	All^*b*^	16 (18)	0.16	6.15	0.02	6.63	0.00	7.40
	1980	6	0.40	5.11	0.27	5.62	0.00	6.96
	1981	12	0.11	6.61	0.00	7.09	0.00	7.61
Notes: CARB dichot, California Air Resources Board dichotomous sampler monitoring; CASTNet, Clean Air Status and Trends Network; CHS, Children’s Health Study; FRM, Federal Reference Method; IMPROVE, Interagency Monitoring of Protected Visual Environment; IPN, Inhalable Particulate Network; PM_2.5_, fine particulate matter; RMSE, root mean square error; WBAN, Weather Bureau Army Navy. ^***a***^Number of sites (number of observations when different from the number of sites). ^***b***^Annual averages for 1994–2003 from CHS, for 1988–2001 from CARB dichot, and for 1980–1981 from IPN.								

**Figure 4:**
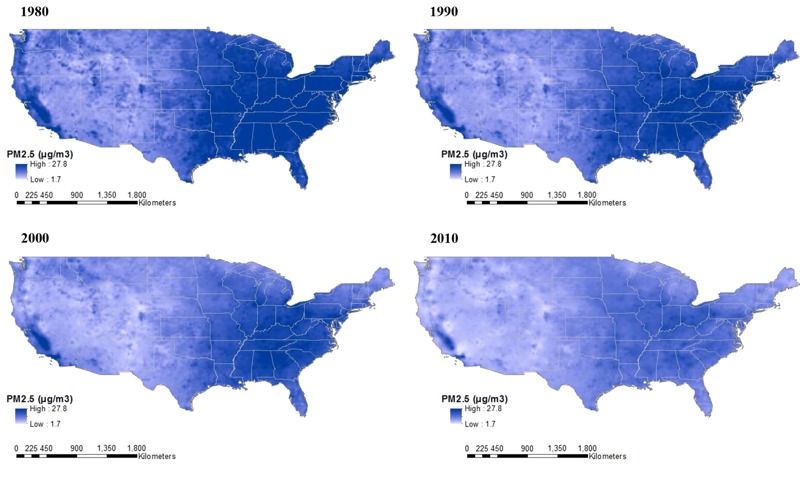
Predicted fine particulate matter (PM_2.5_) annual averages in 1980, 1990, 2000, and 2010 from the 31-year PM_2.5_ model using the extrapolated temporal trend based on PM_2.5_ data for 1999–2010; Maps generated using model outputs discussed in the “Development of the PM_2.5_ model for 1980–2010” in “Methods” and boundaries for the year 2000 U.S. Census. Source: ArcUSA; U.S. Census; ESRI (Pop2010 fields); and ESRI, derived from Tele Atlas. Maps were created using ArcGIS® software by Esri. ArcGIS® and ArcMap™ are the intellectual property of Esri and are used herein under license. Copyright © Esri. All rights reserved. For more information about Esri® software, please visit www.esri.com.

**Figure 5:**
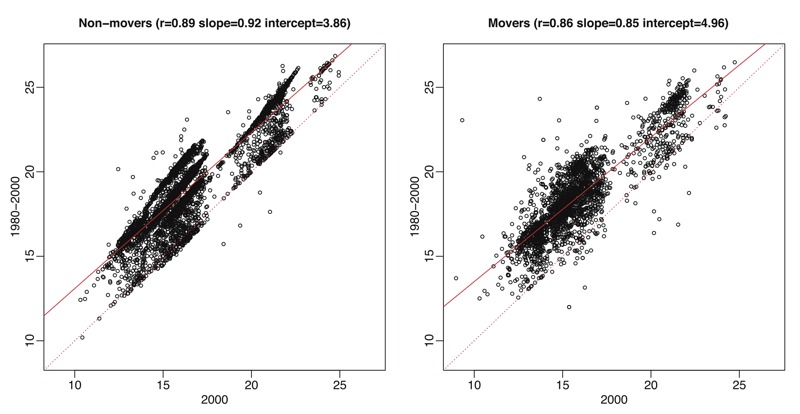
Scatter plots of predicted fine particulate matter (PM_2.5_) annual averages from the 31-year PM_2.5_ model using the extrapolated temporal trend based on PM_2.5_ data for 1999–2010 for 2000 versus long-term averages for 1980–2000 weighted by times of residence across home addresses of 5,086 participants who never moved during 1980–2000 and 2,466 Multi-Ethnic Study of Atherosclerosis (MESA)/MESA Air participants who moved at least once.

Figure 4 shows that the predicted PM_2.5_ concentrations decreased dramatically across decennial years from 1980 through 2010, with only a few areas remaining consistently high in the continental United States over all three decades. The decreasing trend was also clear over 31 years across the 10 most highly populated grid coordinates in each region (data not shown). Thirty-one-year, residence-weighted average PM_2.5_ predictions for MESA Air participants were generally higher than the corresponding annual averages at their residences in 2000 (Figure 5; see also Figure S10). The two sets of predictions showed high correlations with 2000 annual averages (0.86–0.89) with slightly lower correlation and more attenuated slopes for movers than for nonmovers.

## Discussion

We developed a 31-year prediction model to estimate fine-scale ambient PM_2.5_ concentrations in the continental United States, including the time period before 1999, when extensive monitoring data became available. Key aspects of our approach to historical (pre-1999) prediction were our consideration of various trend estimation approaches and validation of our model with multiple external validation data sets. Although the prediction model performed well for 1999–2010 as assessed by cross-validation, the pre-1999 external validation is a more important indicator for evaluating historical predictions. We found that the pre-1999 predictions also generally performed well across three trend estimation approaches, particularly for the external IMPROVE and CHS data. The model performance was better in the highly populated East region. Twenty-one-year average PM_2.5_ concentrations for 1980–2000 at MESA/MESA Air participant residences tended to be higher than and somewhat unsystematically different from annual averages in 2000, although the correlation was higher among those with stable residence locations.

Developing a prediction model for estimating long-term PM_2.5_ concentrations for the time period for which few PM_2.5_ monitoring data are available required using external information to estimate a temporal trend. Our three approaches for trend estimation gave consistently good model performance as assessed by *R* ^2^ values, with a slight edge to the linearly extrapolated trend for predictions before 1990; this may be the case because the three trends we considered, although based on three different data sources, all showed similarly decreasing patterns with only slightly different shapes. We considered PM_2.5_ sulfate data useful for trend estimation because a large reduction of PM_2.5_ in the 1990s and early 2000s was likely to be the result of a large reduction of sulfate, particularly in the East region (Malm et al. 2002; U.S. EPA 2004a). The nonlinear decrease of the estimated trend for the PM_2.5_ sulfate data could have been caused by the timing of the implementation of policies regulating sulfur dioxide emissions (Xing et al. 2013). The decreasing trend for annual sulfur dioxide emissions from power plants matches well with that for sulfate concentrations in the eastern half of the United States between 1990 and 2003 (U.S. EPA 2004a). The CASTNet sites were located mostly in rural areas, which may not represent PM_2.5_ concentrations from urban sources or population centers. However, because sulfate is an important regional pollutant that exhibits homogenous concentrations on a large spatial scale owing to long-range transport, the rural sites allow us to assess large regional trends over time, as intended by the CASTNet monitoring design. The trend estimated from the visibility data had a somewhat different shape from that of the PM_2.5_ sulfate trend, which could possibly be driven by meteorological influence (Hand et al. 2014). In addition to a nonlinear relationship between PM_2.5_ concentrations and visibility depending on chemical composition and weather conditions, the change of sampling methods for visibility (beginning in 1992) from the relatively subjective human eye to more objective optical instruments (Hyslop 2009; U.S. EPA 2005) coincides with the observed state of a marked downward trend.

Our historical model was based on a spatio-temporal framework using annual averages of PM_2.5_ concentrations for multiple years. Other studies in Europe and Canada predicted annual averages of nitrogen dioxide (NO_2_), nitrogen oxides (NO_X_), and PM_2.5_ by back-extrapolation (Beelen et al. 2014; Chen et al. 2010; Gulliver et al. 2013; Meng et al. 2015). The back-extrapolation approach computed the difference of spatial averages between the two time periods or the ratio of a short-term average to an annual average based on a few fixed site measurements and then added to or multiplied by predictions for recent years to obtain estimates for early years. In contrast with the back-extrapolation approach, our spatio-temporal approach allows prediction for an extended time period for which there are no measurements.

As other authors have done, we considered various alternative approaches to historical prediction. Most previous studies used ratios of PM_2.5_ to PM_10_ (particulate matter) to leverage PM_10_ data collected before PM_2.5_ monitoring began, as opposed to our approach, which directly used PM_2.5_ along with an estimated temporal trend. Some U.S. investigators developed ratio models that predicted monthly averages of PM_2.5_ concentrations for 1988–1998 by multiplying the ratios by the predicted concentration of PM_10_ for Nurses’ Health Study participants residing in Northeastern and Midwestern regions (Paciorek et al. 2009; Yanosky et al. 2009) and expanding the model to the continental United States (Yanosky et al. 2014). In Taipei, Taiwan, another study developed a ratio model for predicting historical monthly averages of PM_2.5_ (Yu and Wang 2010). In separate analyses that aimed to mimic this approach, we also applied our model to annual average ratios. Our cross-validated *R* ^2^s were high between 1999 and 2010 (*R* ^2^ = 0.84–0.90), consistent with those of our original model. However, the *R* ^2^ values for the out-of-sample validation using IMPROVE data were lower, particularly in early years such as 1990 and 1991 (*R* ^2^ = 0.13 and 0, respectively). This poor model performance could be attributed to the relatively poor prediction performance of PM_10_ rather than PM_2.5_. A spatio-temporal prediction model for PM_10_ annual averages in the continental United States achieved a cross-validated *R* ^2^ of 0.55 (Hart et al. 2009), much lower than the cross-validated *R* ^2^ of 0.88 in a spatial prediction model for PM_2.5_ annual averages in 2000 (Sampson et al. 2013). It is also possible that PM_10_ temporal and spatial patterns vary differently from those of PM_2.5_.

In addition to ratios, we also explored modeling approaches that incorporated visibility or meteorology to predict historical PM_2.5_ concentrations. A group of studies used the extinction coefficient, the inverse visual range multiplied by a constant, solely or jointly with PM_2.5_ and PM_10_ data based on its high correlation with PM_2.5_ concentrations (Ozkaynak et al. 1985; Paciorek et al. 2009; Yanosky et al. 2009). The good performance we obtained when using the visibility trend in our model confirmed the usefulness of visibility data for predicting PM_2.5_. However, we observed slightly better model performance when using PM_2.5_ data than when using visibility data when validated on the national scale using IMPROVE data. We examined our models after adding meteorological measurements as spatio-temporal covariates and found worse model performance than with our preferred approach.

We evaluated our historical prediction model using four available external validation data sets; together, these covered 13 years of the 19-year period from 1980 to 1998 in much of the United States. Previous studies for historical PM_2.5_ prediction models either presented cross-validated results using data from before 1999 but no external validation data sets (Paciorek et al. 2009; Yanosky et al. 2009, 2014), or they reported external validation results based on a limited data set for a short time period (Hogrefe et al. 2009; Lall et al. 2004; Ozkaynak et al. 1985; Yu and Wang 2010). Our model performed particularly well when evaluated against IMPROVE and CHS data. One strength of using the IMPROVE data as a validation data set is that it is national. The IMPROVE data yielded the highest *R* ^2^ values among all external validation data sets, possibly owing to its advantage of validating for the 1990–1998 time period, when the estimated trend was less uncertain.

We also observed consistently high *R* ^2^s when validating against the data from CHS, which deployed monitoring sites in urban and residential areas. All CHS monitoring sites were in southern California and thus may not be generalizable across the United States. The CARB dichot data, which were also restricted to California locations, gave lower *R* ^2^s, including values < 0.5 for some years. These low *R* ^2^ estimates could have resulted from the lower between-site variability in California (vs. the entire United States) as well as the small number of sites, a few of which had poor predictions. Another possible reason for this poor performance is that the CARB dichot network used a different sampling protocol from that used by FRM. Our simplified data-driven calibration method may not have performed well when compared with an approach incorporating site-specific meteorological conditions (Blanchard et al. 2011). Model performance may have also been affected by a set of CARB dichot sites in the highest PM_2.5_ concentration areas (Figure 4). The IPN data yielded the lowest *R* ^2^s overall, possibly driven by the limited number of IPN sites and the inconsistency between the IPN and FRM sampling protocols. With 6 and 12 sites for 1980 and 1981, respectively, a few sites with poor predictions had a large impact on the *R* ^2^ estimates. Furthermore, the IPN years of 1980–1981 are the earliest years of our prediction period and may reflect the most uncertainty in trend estimation.

This study includes some limitations and implications for future research. We used time-constant geographic variables, which do not account for changes in spatial characteristics over time. However, among the ~300 geographic variables that we used for estimating PLS predictors were two sources of land use data: land cover data created in the 1970s and 1980s and satellite land use imaginary data generated in 2007. These two data sets represent spatial differences in land use in two different time periods separated by ~30 years, and modeling the temporal trend with these covariates incorporated enabled us to capture changes in land use features over time in our model. In addition, a study in Vancouver, Canada, found that their model performance in predicting NO and NO_2_ in 2003 was consistent with geographic variables collected between 2003 and 2010 (Wang et al. 2013). Although this time period is only 7 years and therefore, is much shorter than our 31 years, these findings suggest that spatial patterns in urban areas with stable physical environments can be characterized by geographic variables from one of many time periods. Some previous studies have used aerosol optical depth (AOD) data to improve prediction models for PM_2.5_ (Beckerman et al. 2013; Hystad et al. 2012; Kloog et al. 2011). These models used short-term or long-term averages of AOD. Future studies should investigate how to incorporate AOD measurements into spatio-temporal prediction models for extended time periods and whether the addition of AOD improves the model’s performance.

As with application of any predicted exposure to health analyses, using predicted PM_2.5_ concentrations from our historical prediction model may affect the estimates in subsequent health analyses because of exposure measurement error. As others have shown, we note that the high *R* ^2^ values we obtained do not guarantee the accuracy or proper coverage of health effect estimates owing to Berkson- and classical-like measurement error (Szpiro et al. 2011a). Several simulation studies have shown that exposure models that perform well can still produce biased and/or imprecise health effect estimates (Alexeeff et al. 2015; Szpiro et al. 2011b). One possible explanation for this occurrence is that the monitor locations do not represent the study population locations, resulting in monitored exposures that are spatially incompatible with the population’s exposures (Szpiro and Paciorek 2013).

Our results suggest the importance of incorporating changes in air pollution concentrations in cohort studies. We showed that long-term PM_2.5_ prediction averages for 31 years that incorporated mobility were systematically higher than 2000 predictions among nonmovers and were nonsystematically different in movers. This pattern varied by city, as suggested by Figure S10, possibly depending on the extent of exposure contrast and on the population’s mobility between low- and high-exposure areas within a city. Using exposure predictions from a later period of follow-up in epidemiological study, as is commonly done (Beelen et al. 2008; Cesaroni et al. 2013), may not adequately represent long-term exposures and might have an impact on health effect findings.

## Conclusions

Our 31-year national PM_2.5_ prediction model can be widely applicable to epidemiological studies, particularly for assessing associations between long-term air pollution exposure and health outcomes in cohort studies. Although unavoidable uncertainty about the quality of predictions for the earliest time periods remains, the overall strong performance of our model assures that good PM_2.5_ estimates that are temporally well aligned with health data can be provided, including for health outcomes collected before extensive monitoring data exist. In addition, application of this point-wise prediction model will allow estimation of individual-level concentrations across historical addresses over time and thus will improve assessment of the impact of air pollution on the progression of disease conditions over an individual’s life-course. Our findings also suggest that long-term average PM_2.5_ estimates obtained from single addresses or from restricted time periods after health observation may not accurately represent long-term average estimates for some people and could have an impact on subsequent health analyses.

## Supplemental Material

(492 KB) PDF
