# Supplementary material for: Historical Prediction Modeling Approach for Estimating Long-Term Concentrations of PM2.5 in Cohort Studies before the 1999 Implementation of Widespread Monitoring
Source: Environ Health Perspect. 2016 Jun 24;125(1):38–46. doi: 10.1289/EHP131 (PMC5226688; doi:10.1289/EHP131)
Supplement: (492 KB) PDF [file EHP131.s001.acco.pdf]

Environ Health Perspect
DOI: 10.1289/EHP131

Note to readers with disabilities: *EHP* strives to ensure that all journal content is accessible to all readers. However, some figures and Supplemental Material published in *EHP* articles may not conform to 508 standards due to the complexity of the information being presented. If you need assistance accessing journal content, please contact ehp508@niehs.nih.gov. Our staff will work with you to assess and meet your accessibility needs within 3 working days.

Supplemental Material

Historical Prediction Modeling Approach for Estimating Long-Term Concentrations of $PM_{2.5}$ in Cohort Studies before the 1999 Implementation of Widespread Monitoring

Sun-Young Kim, Casey Olives, Lianne Sheppard, Paul D. Sampson, Timothy V. Larson, Joshua P. Keller, and Joel D. Kaufman

Table of Contents

Table S1. List of geographic variables

Table S2. Cross-validation statistics of the historical $PM_{2.5}$ models for 1999-2010 by year and region

Table S3. Regression coefficients of cross-validated predictions against observations from the historical $PM_{2.5}$ model by year and three regions from 1999 through 2010

Table S4. Proportion of total variance of the cross-validated predictions captured by the long-term mean, the temporal trend, and spatio-temporal residuals across FRM and IMPROVE sites

Table S5. Regression coefficients of predictions against observations from the historical $PM_{2.5}$ model using IMPROVE data for 1990-1998 by year and region

Table S6. Regression coefficients of predictions against observations from the historical $PM_{2.5}$ models using CHS, CARB dichot, and IPN data by year

Figure S1. Number of monitoring sites for $PM_{2.5}$ in FRM and IMPROVE from 1999 through 2010

Figure S2. Time-series plots of annual averages for $PM_{2.5}$ across FRM and IMPROVE sites for 1999-2010 by region

Figure S3. Estimated regression and variance parameters of the $PM_{2.5}$ prediction model for 1980-2010

Figure S4. Loadings of geographic variables for two PLS predictors by the long-term mean and trend coefficient

Figure S5. Scatter plots of observed and predicted $PM_{2.5}$ annual averages from the $PM_{2.5}$ historical model using the FRM/IMPROVE $PM_{2.5}$ trend across CHS sites for 1994-2003

Figure S6. Scatter plots of observed and predicted $PM_{2.5}$ annual averages from the $PM_{2.5}$ historical model using the FRM/IMPROVE $PM_{2.5}$ trend across CARB dichot sites for 1988-2001

Figure S7. Scatter plots of observed and predicted $PM_{2.5}$ annual averages from the $PM_{2.5}$ historical model using the FRM/IMPROVE $PM_{2.5}$ trend across IPN sites for 1980-1981

Figure S8. Boxplots and spaghetti plots of differences between maximum and minimum of predicted $PM_{2.5}$ annual averages across three trend estimation approaches over years at IMPROVE sites

Figure S9. Maps of differences between maximum and minimum of predicted $PM_{2.5}$ annual averages across three trend estimation approaches at IMPROVE sites in 1980, 1985, 1990 and 1998

Figure S10. Scatter plots of predicted $PM_{2.5}$ annual averages from the 31-year $PM_{2.5}$ model using the extrapolated temporal trend based on $PM_{2.5}$ data for 1999-2010 for 2000 vs. long-term averages for 1980-2000 weighted by times of residences across home addresses of 5,086 participants who never moved for 1980-2000 and 2,466 MESA/MESA Air participants who moved at least once by six MESA metropolitan areas

Table S1. List of geographic variables

Category	Measure	Variable description
Traffic	Distance to the nearest road	Any road, A1, intersection
	Sum within buffers of 0.05-15 km	A1, A2+A3, truck route, intersections
Population	Sum within buffers of 0.5-3 km	Population in block groups
Land use 1970-80s	Percent within buffers of 0.05-15 km	Urban or Built-Up land
(urban)		(residential, commercial, industrial, transportation, urban)
(rural)		Agricultural land (cropland, groves, feeding)
		Rangeland (herbaceous, shrub)
		Forest land (green, forest, mixed forest)
		Water (streams, lakes, reservoirs, bays)
		Wetland
		Barren land (beaches, dry salt flats, sand, mines, rock)
		Tundra
		Perennial snow or ice
Land use 2006	Percent within buffers of 0.05-15 km	Urban or Built-Up land
(urban)		(developed low, medium, and high density, open space)
(rural)		Agricultural land (cropland)
		Rangeland (shrub, grass, pasture)
		Forest land (deciduous forest, mixed forest, evergreen)
		Water (water)
		Wetland
		Barren land
		Perennial snow or ice
Position	Coordinates	Longitude, latitude
Source	Distance to the nearest source	Coastline, Coastline (rough)
		Commercial area
		Railroad, Railyard
		Airport

		Major airport<br>Large port<br>City hall
Emission	Sum within buffers of 3-30 km	$PM_{2.5}$<br>PM_{10}<br>CO<br>SO_2<br>NO_x
Vegetation	Quantiles within buffers of 0.5-10 km	Normalized Difference Vegetation Index (NDVI)
Imperviousness	Percent within buffers of 0.05-5 km	Impervious surface value
Elevation	Elevation above sea levels<br>Counts of points above or below a threshold within buffers of 1-5 km	Elevation value
Residual oil	Distance to the nearest boiler<br>Sum within buffers of 0.1-3 km	Residual oil grade 4 or 6<br>Total residual oil active heating capacity

Table S2. Cross-validation statistics of the historical $PM_{2.5}$ models for 1999-2010 by year and region

Estimated trend		FRM/IMPROVE[a] $PM_{2.5}$		CASTNet[a] $PM_{2.5}$ sulfate		WBAN[a] visibility	
Cross-validation statistics		R^2	RMSE (μg/m^3)	R^2	RMSE (μg/m^3)	R^2	RMSE (μg/m^3)
Year/region	N[b]						
All[c]	1,460 (10,800)	0.87	1.44	0.86	1.46	0.86	1.50
1999	523	0.86	1.81	0.86	1.81	0.85	1.86
2000	865	0.85	1.54	0.85	1.55	0.85	1.56
2001	988	0.86	1.52	0.86	1.52	0.86	1.53
2002	1,054	0.84	1.55	0.84	1.56	0.84	1.57
2003	969	0.85	1.46	0.84	1.48	0.84	1.50
2004	980	0.86	1.41	0.85	1.43	0.85	1.46
2005	940	0.88	1.44	0.88	1.46	0.87	1.50
2006	898	0.86	1.37	0.85	1.39	0.84	1.43
2007	937	0.86	1.36	0.86	1.39	0.85	1.44
2008	902	0.82	1.35	0.81	1.38	0.79	1.45
2009	884	0.80	1.27	0.79	1.32	0.77	1.38
2010	860	0.83	1.27	0.81	1.35	0.80	1.41
East[c]	1,056 (7,956)	0.86	1.09	0.86	1.12	0.86	1.12
Mountain West[c]	239 (1,594)	0.59	1.96	0.59	1.98	0.60	1.94
West Coast[c]	165 (1,250)	0.84	2.35	0.84	2.35	0.80	2.57
All (by site)[d]	11	0.58	0.85	0.55	0.88	0.57	0.89

a. FRM = Federal Reference Method; IMPROVE = Interagency Monitoring of Protected Visual Environment; CASTNet = Clean Air Status and Trends Network; WBAN = Weather-Bureau-Army-Navy

b. Number of sites (Number of observations when different from the number of sites)

c. Annual averages from 1999 through 2010

d. Median number of observations and median of cross-validation statistics at each site where there are more than 6 years of data

Table S3. Regression coefficients of cross-validated predictions against observations from the historical $PM_{2.5}$ model by year and three regions from 1999 through 2010

Data for trend estimation		FRM/IMPROVE $PM_{2.5}$		CASTNET $PM_{2.5}$ sulfate		WBAN visibility	
Coefficients		Slope (95% CI)	Intercept (95% CI)	Slope (95% CI)	Intercept (95% CI)	Slope (95% CI)	Intercept (95% CI)
Year/region	N						
All	1,460	0.84 (0.84, 0.85)	1.81 (1.74, 1.88)	0.84 (0.84, 0.85)	1.81 (1.73, 1.88)	0.85 (0.85, 0.86)	1.71 (1.63, 1.79)
1999	523	0.82 (0.79, 0.85)	2.42 (2.02, 2.82)	0.82 (0.79, 0.85)	2.47 (2.07, 2.86)	0.83 (0.80, 0.86)	2.28 (1.86, 2.70)
2000	865	0.84 (0.81, 0.86)	2.16 (1.85, 2.47)	0.84 (0.81, 0.86)	2.17 (1.87, 2.48)	0.85 (0.83, 0.87)	1.98 (1.66, 2.29)
2001	988	0.83 (0.81, 0.85)	2.21 (1.94, 2.47)	0.83 (0.81, 0.85)	2.20 (1.93, 2.46)	0.84 (0.82, 0.86)	2.03 (1.76, 2.30)
2002	1,054	0.81 (0.79, 0.84)	2.29 (2.03, 2.54)	0.81 (0.79, 0.83)	2.29 (2.03, 2.55)	0.83 (0.81, 0.85)	2.12 (1.86, 2.39)
2003	969	0.83 (0.81, 0.86)	1.95 (1.69, 2.22)	0.84 (0.81, 0.86)	1.94 (1.67, 2.21)	0.85 (0.82, 0.87)	1.80 (1.52, 2.08)
2004	980	0.83 (0.81, 0.85)	1.90 (1.65, 2.14)	0.84 (0.81, 0.86)	1.86 (1.61, 2.11)	0.85 (0.83, 0.87)	1.73 (1.47, 1.99)
2005	940	0.85 (0.83, 0.87)	1.83 (1.59, 2.08)	0.85 (0.83, 0.87)	1.81 (1.56, 2.06)	0.86 (0.84, 0.88)	1.73 (1.47, 1.99)
2006	898	0.83 (0.80, 0.85)	1.90 (1.66, 2.15)	0.83 (0.81, 0.85)	1.86 (1.61, 2.11)	0.84 (0.82, 0.86)	1.76 (1.50, 2.03)
2007	937	0.83 (0.81, 0.86)	1.89 (1.64, 2.13)	0.83 (0.81, 0.86)	1.88 (1.63, 2.13)	0.84 (0.82, 0.87)	1.79 (1.52, 2.06)
2008	902	0.80 (0.77, 0.82)	2.10 (1.84, 2.35)	0.79 (0.77, 0.82)	2.11 (1.85, 2.37)	0.80 (0.77, 0.83)	2.07 (1.79, 2.35)
2009	884	0.79 (0.76, 0.81)	1.95 (1.71, 2.19)	0.78 (0.75, 0.81)	2.00 (1.75, 2.25)	0.79 (0.76, 0.82)	1.96 (1.69, 2.23)
2010	860	0.82 (0.80, 0.85)	1.66 (1.43, 1.90)	0.81 (0.78, 0.84)	1.77 (1.53, 2.02)	0.82 (0.79, 0.85)	1.71 (1.45, 1.98)
East	1,056	0.88 (0.87, 0.89)	1.40 (1.31, 1.50)	0.87 (0.86, 0.88)	1.50 (1.40, 1.60)	0.88 (0.87, 0.89)	1.40 (1.31, 1.50)
Mountain West	239	0.67 (0.65, 0.70)	2.61 (2.42, 2.79)	0.66 (0.64, 0.69)	2.71 (2.53, 2.90)	0.66 (0.63, 0.68)	2.69 (2.51, 2.87)
West Coast	165	0.78 (0.76, 0.80)	2.51 (2.29, 2.74)	0.81 (0.79, 0.83)	2.24 (2.00, 2.47)	0.82 (0.80, 0.85)	2.20 (1.94, 2.47)

Table S4. Proportion of total variance of the cross-validated predictions captured by the long-term mean, the temporal trend, and spatio-temporal residuals across FRM and IMPROVE sites

	Long-term mean (a)	Temporal trend (a)	Spatio-temporal residual (a)
Regression Kriging (including regression)			
0.32	0.84	0.09	0.07

a. Sum of the proportions attributable to the long-term mean, the temporal trend, and spatio-temporal residual is equal to 1

Table S5. Regression coefficients of predictions against observations from the historical $PM_{2.5}$ model using IMPROVE data for 1990-1998 by year and region

Data for trend estimation		FRM/IMPROVE $PM_{2.5}$		CASTNET $PM_{2.5}$ sulfate		WBAN visibility	
Coefficient		Slope (95% CI)	Intercept (95% CI)	Slope (95% CI)	Intercept (95% CI)	Slope (95% CI)	Intercept (95% CI)
Year/region	N						
All	72	0.81 (0.79, 0.83)	1.47 (1.32, 1.62)	0.65 (0.63, 0.67)	2.10 (1.97, 2.22)	0.74 (0.72, 0.76)	2.06 (1.90, 2.22)
1990	30	0.84 (0.73, 0.96)	1.38 (0.66, 2.09)	0.61 (0.52, 0.71)	2.51 (1.92, 3.10)	0.69 (0.57, 0.82)	2.60 (1.80, 3.40)
1991	36	0.84 (0.74, 0.94)	1.69 (1.04, 2.34)	0.64 (0.55, 0.72)	2.49 (1.95, 3.03)	0.74 (0.64, 0.85)	2.63 (1.94, 3.32)
1992	37	0.84 (0.77, 0.91)	1.51 (0.95, 2.08)	0.65 (0.59, 0.72)	2.22 (1.73, 2.71)	0.78 (0.70, 0.86)	2.35 (1.75, 2.95)
1993	45	0.79 (0.74, 0.85)	1.58 (1.14, 2.02)	0.62 (0.57, 0.67)	2.24 (1.86, 2.62)	0.73 (0.67, 0.79)	2.33 (1.86, 2.80)
1994	50	0.81 (0.75, 0.87)	1.26 (0.83, 1.69)	0.64 (0.60, 0.69)	1.90 (1.58, 2.22)	0.75 (0.70, 0.81)	1.89 (1.48, 2.29)
1995	58	0.84 (0.79, 0.89)	1.56 (1.22, 1.91)	0.67 (0.63, 0.71)	2.10 (1.81, 2.40)	0.77 (0.71, 0.82)	2.08 (1.70, 2.45)
1996	56	0.85 (0.80, 0.91)	1.14 (0.76, 1.52)	0.70 (0.65, 0.74)	1.70 (1.39, 2.01)	0.78 (0.73, 0.84)	1.53 (1.15, 1.92)
1997	57	0.80 (0.76, 0.85)	1.39 (1.06, 1.73)	0.66 (0.62, 0.70)	1.93 (1.62, 2.24)	0.73 (0.69, 0.78)	1.75 (1.41, 2.08)
1998	54	0.75 (0.70, 0.81)	1.53 (1.13, 1.92)	0.63 (0.58, 0.68)	2.07 (1.71, 2.43)	0.69 (0.64, 0.74)	1.85 (1.49, 2.21)
East	21	0.77 (0.72, 0.81)	2.15 (1.66, 2.64)	0.65 (0.61, 0.68)	2.20 (1.81, 2.60)	0.73 (0.68, 0.78)	2.40 (1.88, 2.92)
Mountain West	34	0.80 (0.72, 0.87)	1.44 (1.14, 1.74)	0.77 (0.71, 0.84)	1.74 (1.49, 1.99)	0.90 (0.82, 0.98)	1.61 (1.30, 1.92)
West Coast	17	0.63 (0.55, 0.71)	2.35 (1.89, 2.81)	0.54 (0.47, 0.60)	2.33 (1.94, 2.72)	0.57 (0.49, 0.64)	2.46 (2.00, 2.91)

Table S6. Regression coefficients of predictions against observations from the historical $PM_{2.5}$ models using CHS, CARB dichot, and IPN data by year

Validation data	Data for trend estimation		FRM/IMPROVE $PM_{2.5}$		CASTNET $PM_{2.5}$ sulfate		WBAN visibility	
	Coefficient		Slope (95% CI)	Intercept (95% CI)	Slope (95% CI)	Intercept (95% CI)	Slope (95% CI)	Intercept (95% CI)
	Year	N						
CHS	All	13	0.55 (0.51, 0.59)	7.33 (6.68, 7.98)	0.58 (0.54, 0.63)	6.57 (5.74, 7.40)	0.65 (0.60, 0.70)	6.33 (5.50, 7.16)
	1994	12	0.49 (0.38, 0.60)	7.91 (5.68, 10.14)	0.51 (0.41, 0.61)	6.48 (4.49, 8.47)	0.57 (0.46, 0.68)	7.19 (4.99, 9.39)
	1995	12	0.46 (0.39, 0.54)	7.64 (5.99, 9.30)	0.48 (0.40, 0.56)	6.35 (4.71, 7.99)	0.54 (0.45, 0.62)	6.84 (4.99, 8.69)
	1996	12	0.52 (0.44, 0.61)	7.17 (5.62, 8.71)	0.54 (0.45, 0.63)	5.98 (4.27, 7.69)	0.60 (0.49, 0.70)	6.21 (4.17, 8.25)
	1997	12	0.60 (0.51, 0.70)	6.36 (4.73, 7.99)	0.63 (0.53, 0.73)	5.19 (3.42, 6.96)	0.70 (0.58, 0.81)	5.08 (3.06, 7.10)
	1998	12	0.64 (0.54, 0.75)	6.23 (4.57, 7.89)	0.68 (0.57, 0.79)	5.15 (3.35, 6.94)	0.74 (0.62, 0.87)	4.90 (2.84, 6.97)
	1999	12	0.52 (0.42, 0.62)	9.16 (7.43, 10.89)	0.61 (0.50, 0.72)	8.53 (6.58, 10.48)	0.67 (0.54, 0.79)	8.04 (5.81, 10.28)
	2000	12	0.59 (0.49, 0.69)	7.21 (5.54, 8.87)	0.66 (0.54, 0.78)	6.59 (4.61, 8.57)	0.72 (0.58, 0.87)	6.13 (3.75, 8.51)
	2001	12	0.60 (0.48, 0.71)	6.62 (4.39, 8.85)	0.66 (0.52, 0.79)	5.89 (3.33, 8.46)	0.72 (0.56, 0.87)	5.36 (2.35, 8.38)
	2002	12	0.66 (0.54, 0.77)	6.84 (4.92, 8.76)	0.72 (0.58, 0.85)	6.12 (3.88, 8.36)	0.78 (0.62, 0.95)	5.57 (2.88, 8.26)
	2003	12	0.70 (0.58, 0.82)	4.62 (2.75, 6.48)	0.76 (0.62, 0.89)	3.97 (1.88, 6.06)	0.82 (0.66, 0.98)	3.53 (1.02, 6.04)
Dichot	All	33	0.49 (0.43, 0.55)	8.65 (7.31, 9.99)	0.49 (0.43, 0.55)	7.58 (6.27, 8.90)	0.56 (0.49, 0.62)	7.50 (6.13, 8.87)
	1988	8	0.36 (0.13, 0.59)	12.18 (4.80, 19.56)	0.40 (0.18, 0.62)	9.52 (2.61, 16.43)	0.43 (0.20, 0.65)	9.94 (2.86, 17.01)
	1989	12	0.51 (0.29, 0.74)	6.03 (-0.30, 12.36)	0.52 (0.33, 0.71)	4.57 (-0.79, 9.92)	0.57 (0.38, 0.77)	4.51 (-1.02, 10.04)
	1990	11	0.65 (0.52, 0.77)	5.11 (1.92, 8.30)	0.65 (0.55, 0.75)	3.65 (1.13, 6.16)	0.72 (0.59, 0.84)	3.87 (0.76, 6.98)
	1991	12	0.40 (0.24, 0.57)	9.30 (4.71, 13.90)	0.42 (0.28, 0.57)	7.31 (3.23, 11.38)	0.47 (0.31, 0.63)	8.17 (3.62, 12.72)
	1992	14	0.65 (0.36, 0.94)	4.64 (-1.89, 11.17)	0.65 (0.39, 0.90)	3.31 (-2.31, 8.93)	0.74 (0.45, 1.02)	3.58 (-2.84, 9.99)
	1993	15	0.53 (0.30, 0.76)	8.44 (3.29, 13.59)	0.59 (0.36, 0.82)	5.56 (0.37, 10.75)	0.66 (0.41, 0.90)	6.30 (0.83, 11.77)
	1994	13	0.63 (0.47, 0.80)	5.62 (2.15, 9.09)	0.63 (0.47, 0.78)	4.31 (1.04, 7.58)	0.72 (0.56, 0.88)	4.42 (1.00, 7.85)
	1995	12	0.59 (0.35, 0.83)	6.81 (2.44, 11.19)	0.56 (0.35, 0.77)	5.78 (1.85, 9.71)	0.60 (0.35, 0.84)	6.53 (1.98, 11.07)
	1996	15	0.64 (0.36, 0.91)	7.80 (3.14, 12.46)	0.68 (0.41, 0.94)	5.97 (1.46, 10.48)	0.75 (0.45, 1.04)	5.88 (0.87, 10.88)

	1997	15	0.92	(0.61, 1.22)	3.51	(-1.33, 8.34)	0.90	(0.58, 1.22)	2.74	(-2.37, 7.84)	0.96	(0.62, 1.30)	2.45	(-3.00, 7.89)
	1998	16	0.71	(0.32, 1.11)	5.72	(-0.58, 12.02)	0.69	(0.30, 1.08)	5.34	(-0.90, 11.58)	0.74	(0.32, 1.15)	4.97	(-1.66, 11.60)
	1999	12	0.79	(0.63, 0.94)	5.33	(2.33, 8.32)	0.80	(0.63, 0.98)	5.11	(1.70, 8.51)	0.82	(0.63, 1.02)	4.82	(1.05, 8.58)
	2000	6	0.82	(0.41, 1.23)	1.52	(-6.52, 9.56)	0.81	(0.39, 1.23)	1.49	(-6.77, 9.76)	0.82	(0.36, 1.28)	1.28	(-7.77, 10.33)
	2001	3	-0.25	(-2.04, 1.53)	20.56	(-20.25, 61.37)	-0.23	(-2.10, 1.63)	20.21	(-22.35, 62.78)	-0.27	(-2.15, 1.60)	21.40	(-21.42, 64.22)
IPN	All	16	0.23	(0.00, 0.47)	14.51	(9.33, 19.70)	0.19	(-0.02, 0.39)	14.38	(9.84, 18.93)	0.20	(0.01, 0.38)	12.49	(8.37, 16.60)
	1980	6	0.34	(-0.08, 0.76)	11.69	(2.62, 20.76)	0.28	(-0.08, 0.64)	12.11	(4.34, 19.89)	0.27	(-0.02, 0.56)	9.96	(3.67, 16.25)
	1981	12	0.18	(-0.11, 0.48)	15.88	(9.15, 22.61)	0.15	(-0.12, 0.41)	15.46	(9.47, 21.44)	0.16	(-0.08, 0.39)	13.85	(8.53, 19.18)

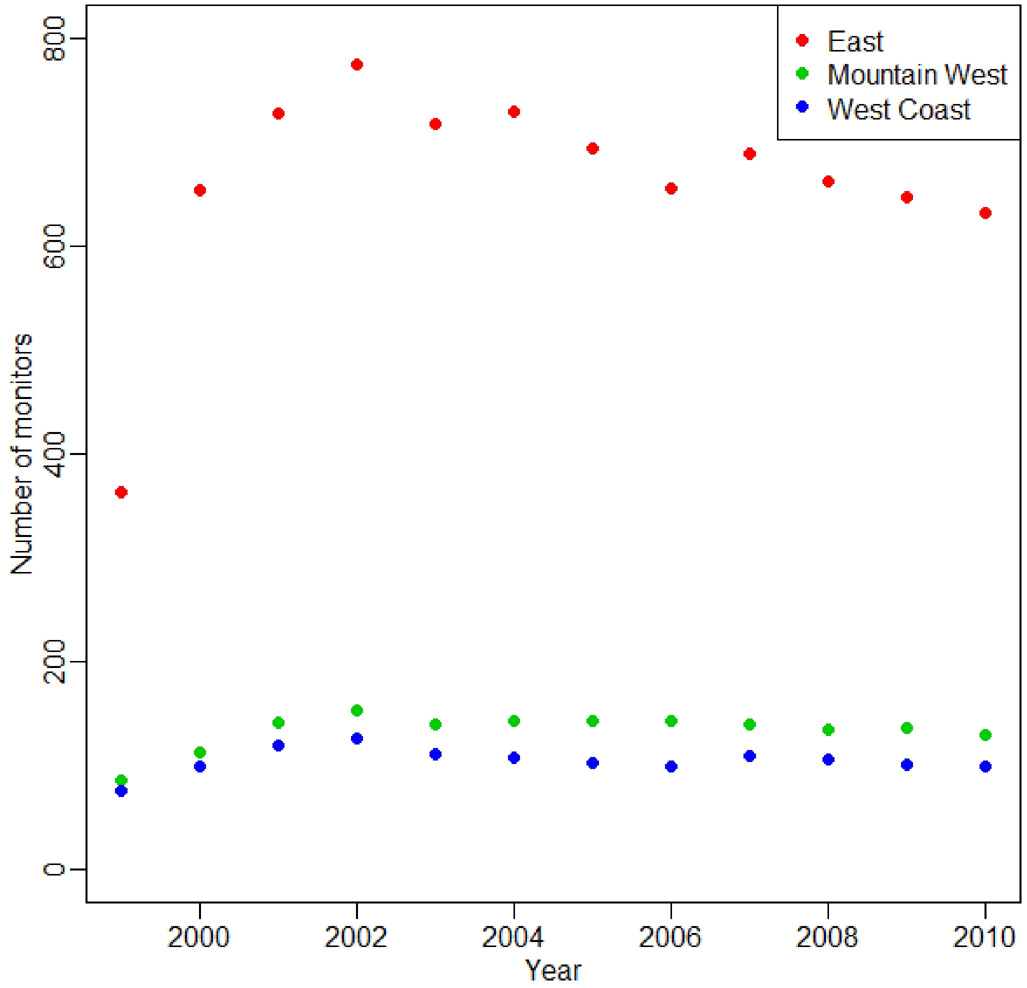

Figure S1. Number of monitoring sites for $PM_{2.5}$ in FRM and IMPROVE from 1999 through 2010

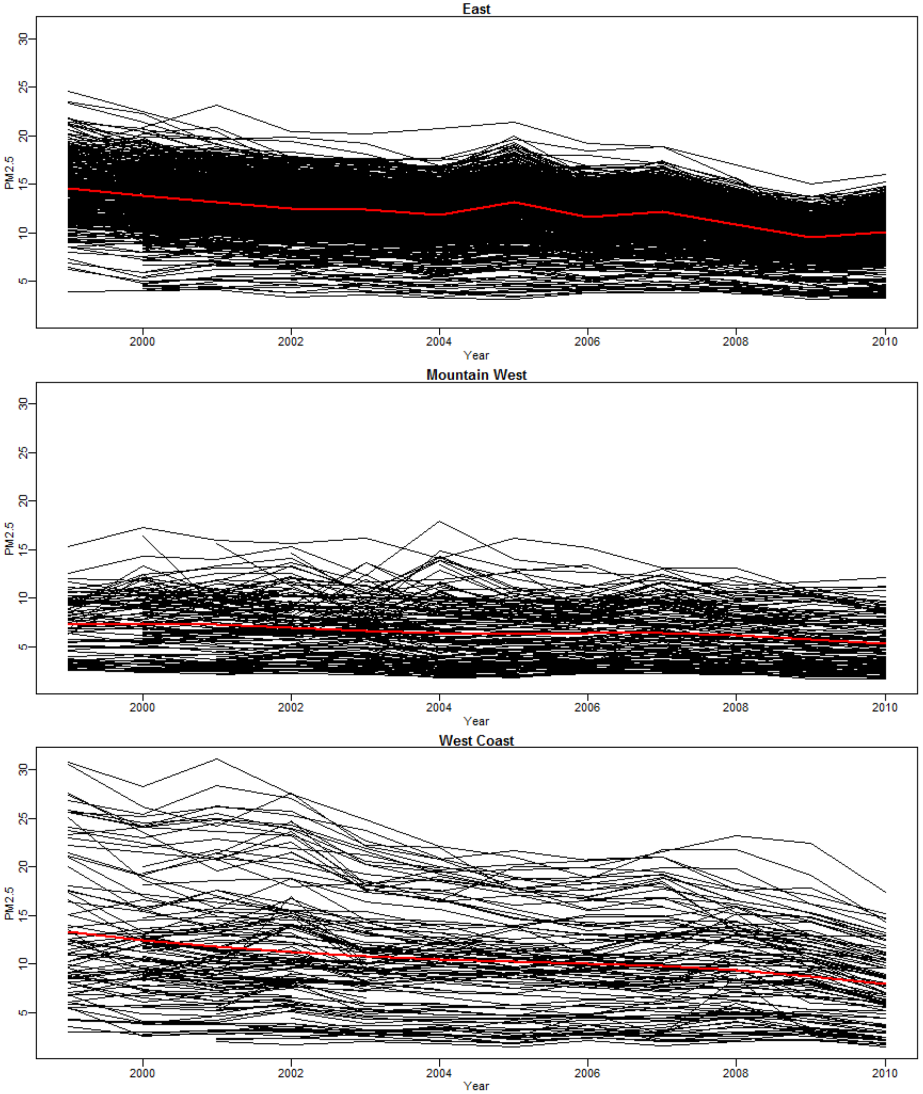

Figure S2. Time-series plots of annual averages for $PM_{2.5}$ across FRM and IMPROVE sites for 1999-2010 by region

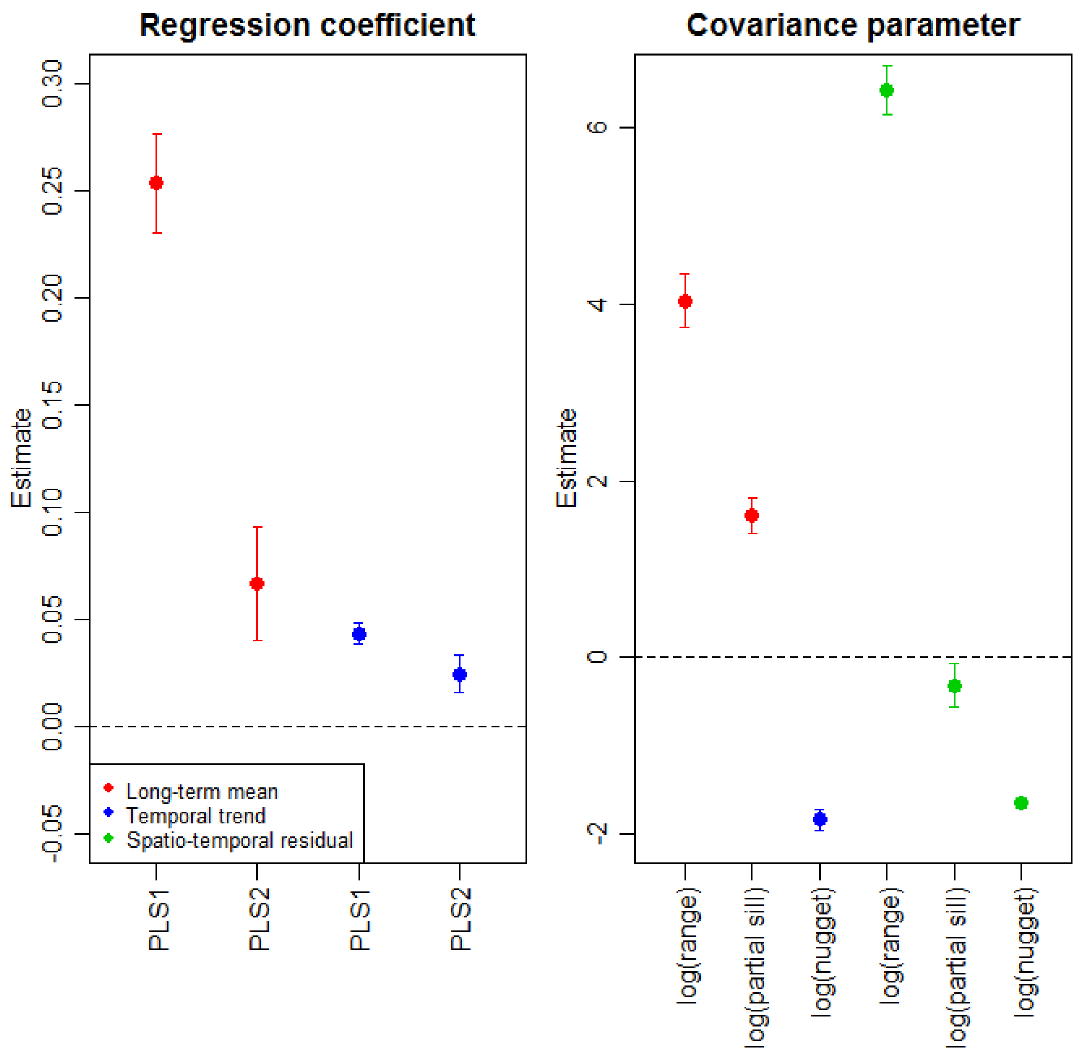

Figure S3. Estimated regression and variance parameters of the $PM_{2.5}$ prediction model for 1980-2010

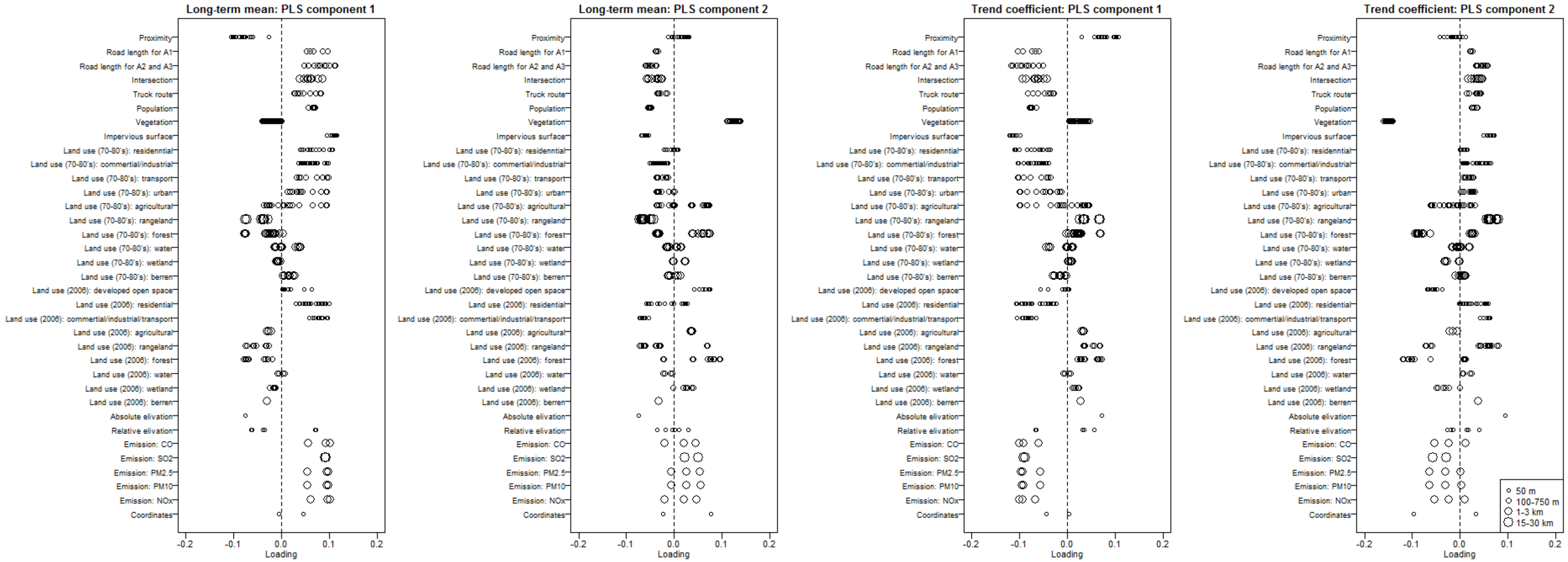

Figure S4. Loadings of geographic variables for two PLS predictors by the long-term mean and trend coefficient

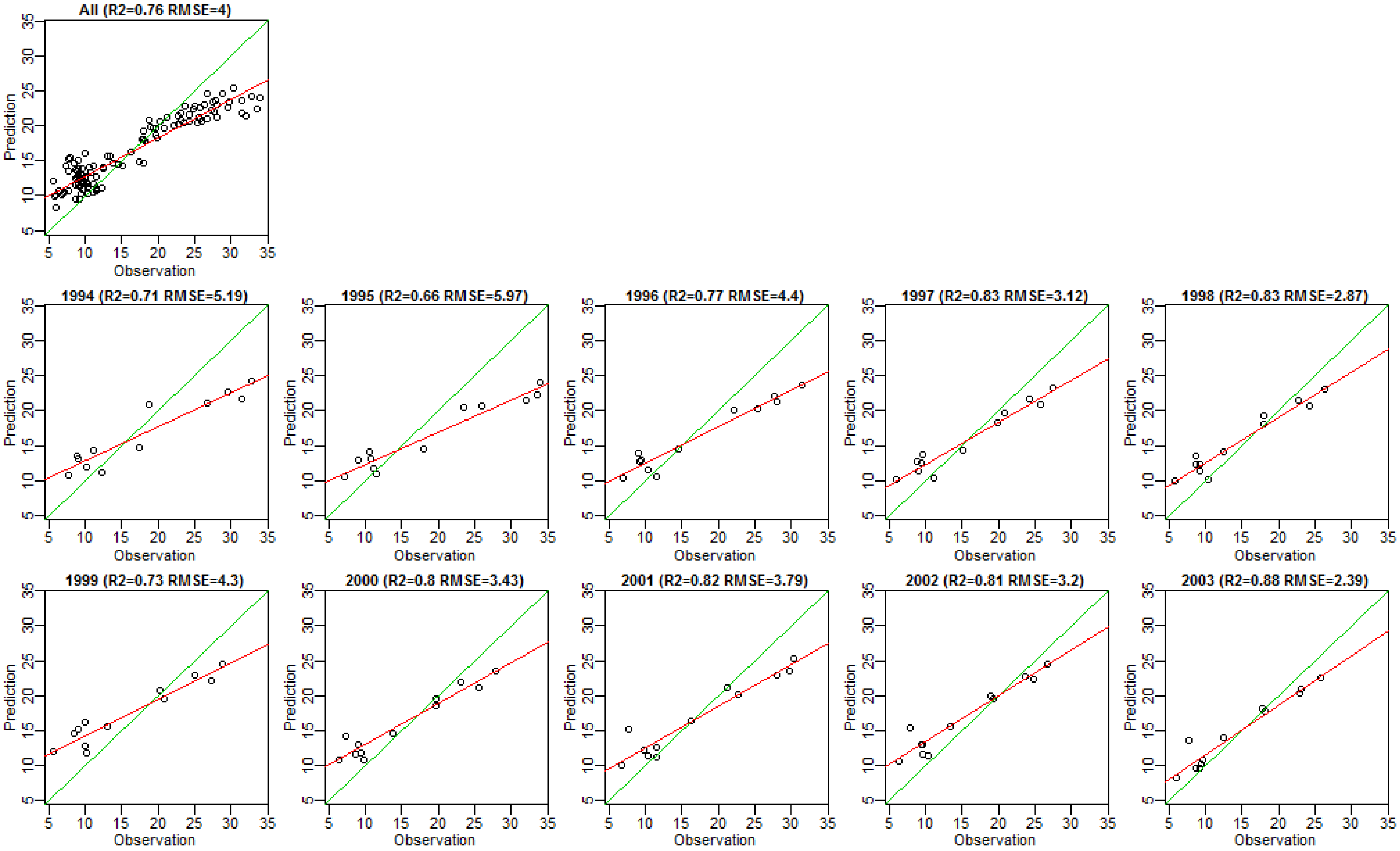

Figure S5. Scatter plots of observed and predicted $PM_{2.5}$ annual averages from the $PM_{2.5}$ historical model using the FRM/IMPROVE $PM_{2.5}$ trend across CHS sites for 1994-2003

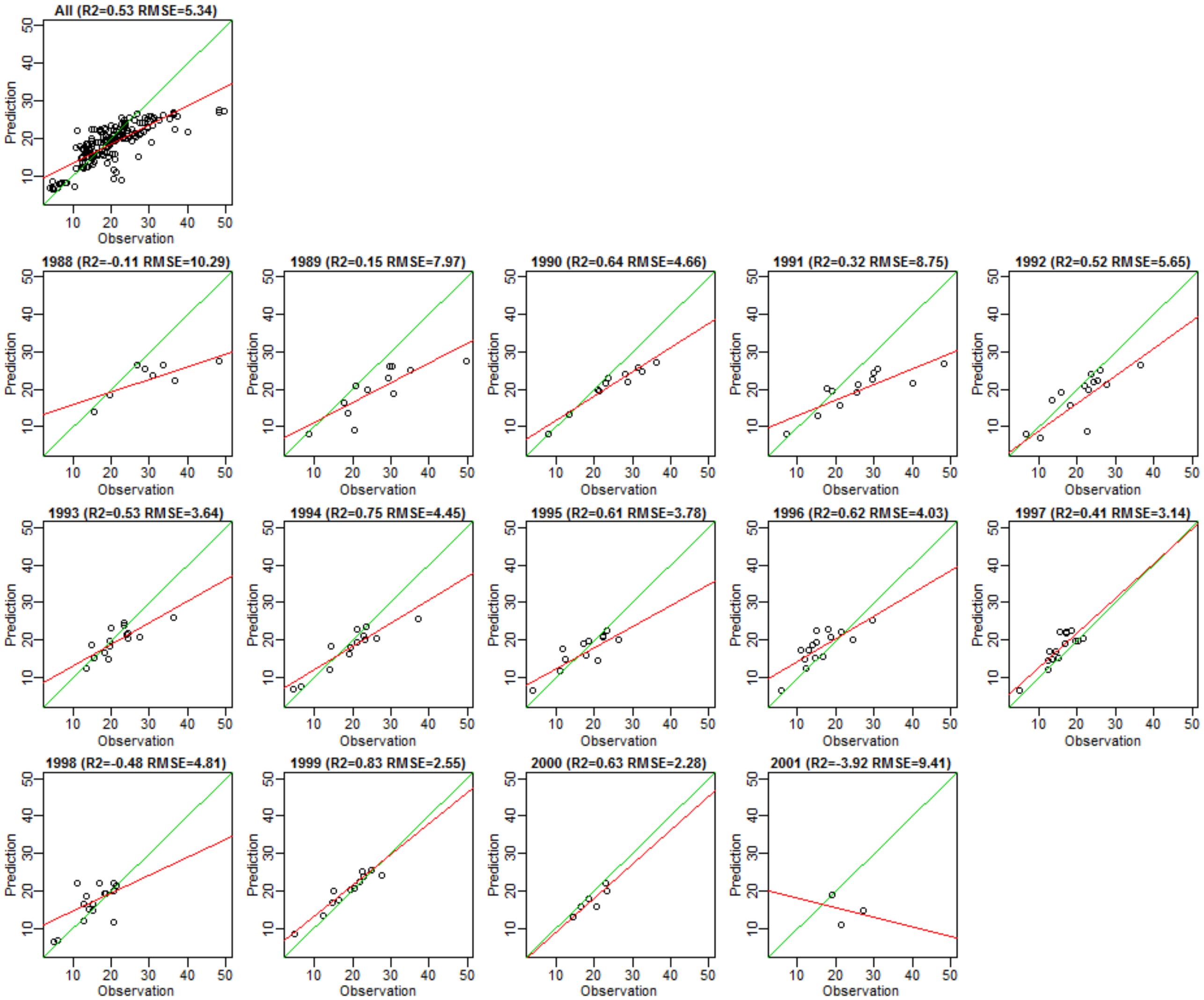

Figure S6. Scatter plots of observed and predicted $PM_{2.5}$ annual averages from the $PM_{2.5}$ historical model using the FRM/IMPROVE $PM_{2.5}$ trend across CARB dichot sites for 1988-2001

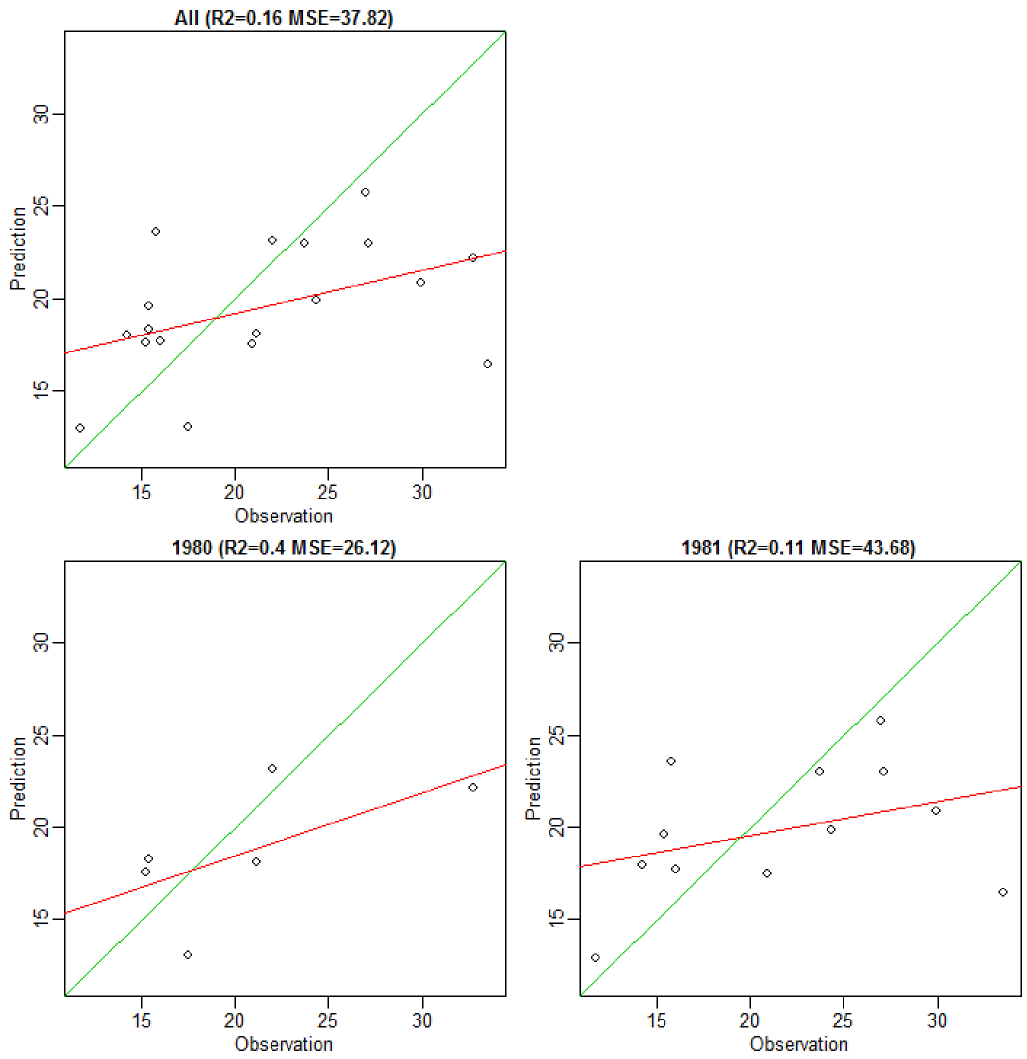

Figure S7. Scatter plots of observed and predicted $PM_{2.5}$ annual averages from the $PM_{2.5}$ historical model using the FRM/IMPROVE $PM_{2.5}$ trend across IPN sites for 1980-1981

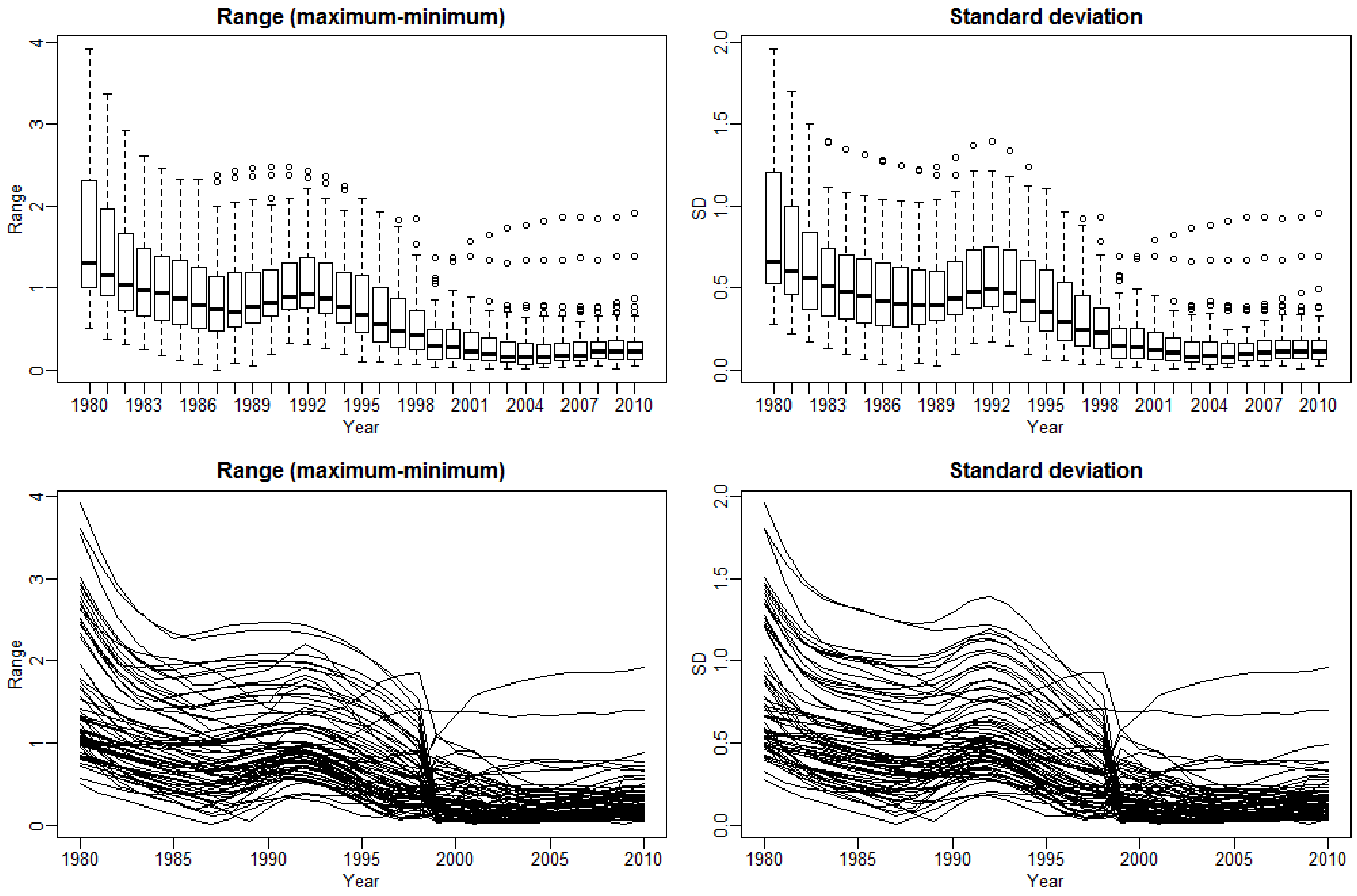

Figure S8. Boxplots and spaghetti plots of differences between maximum and minimum of predicted $PM_{2.5}$ annual averages across three trend estimation approaches over years at IMPROVE sites

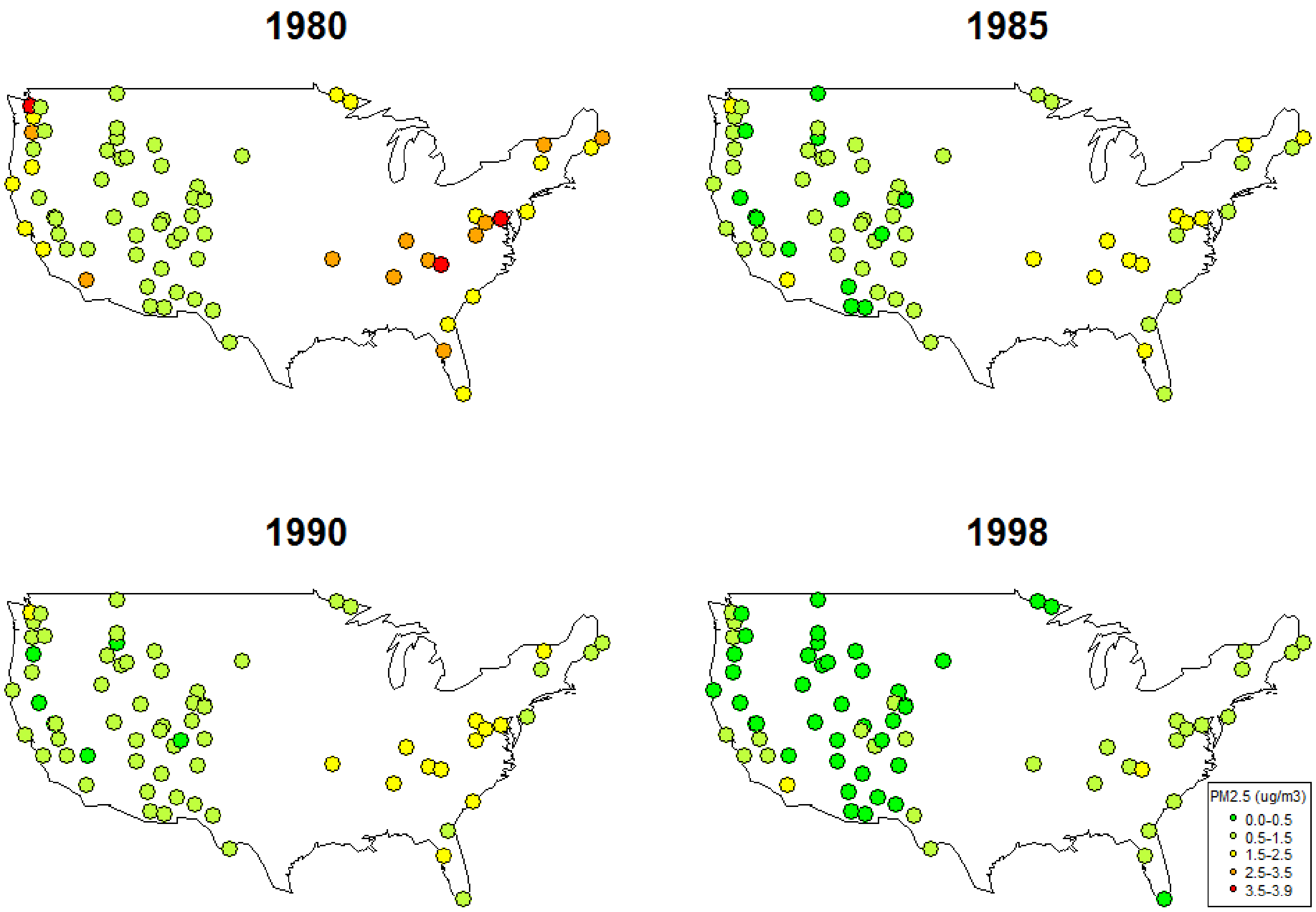

Figure S9. Maps of differences between maximum and minimum of predicted $PM_{2.5}$ annual averages across three trend estimation approaches at IMPROVE sites in 1980, 1985, 1990 and 1998

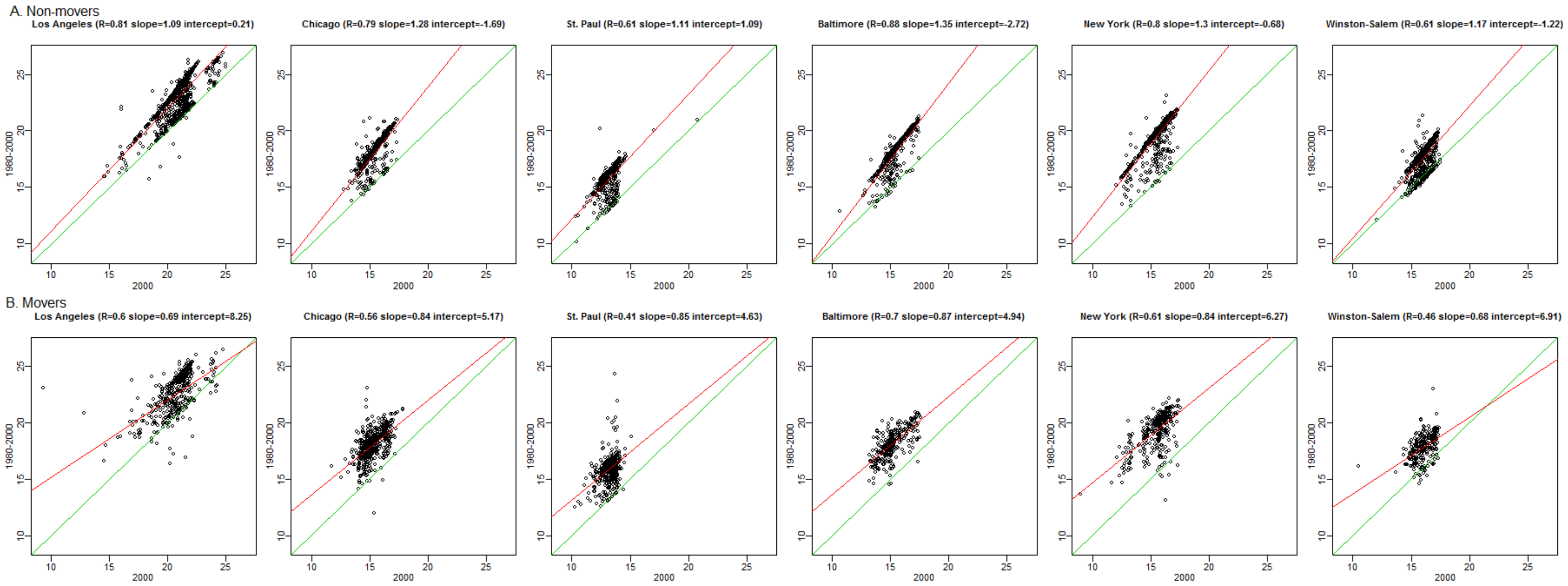

Figure S10. Scatter plots of predicted $PM_{2.5}$ annual averages from the 31-year $PM_{2.5}$ model using the extrapolated temporal trend based on $PM_{2.5}$ data for 1999-2010 for 2000 vs. long-term averages for 1980-2000 weighted by times of residences across home addresses of 5,086 participants who never moved for 1980-2000 and 2,466 MESA/MESA Air participants who moved at least once by six MESA metropolitan areas
